# Entire solutions of nonlinear differential-difference equations

**DOI:** 10.1186/s40064-016-2255-9

**Published:** 2016-05-12

**Authors:** Cuiping Li, Feng Lü, Junfeng Xu

**Affiliations:** College of Science, China University of Petroleum, Qingdao, 266580 Shandong People’s Republic of China; Department of Mathematics, Wuyi University, Jiangmen, 529020 Guangdong People’s Republic of China

**Keywords:** Difference equation, Meromorphic function, Logarithmic order, Nevanlinna theory, Difference polynomials, 30D35, 39B12

## Abstract

In this paper, we describe the properties of entire solutions of a nonlinear differential-difference equation and a Fermat type equation, and improve several previous theorems greatly. In addition, we also deduce a uniqueness result for an entire function *f*(*z*) that shares a set with its shift $f(z+c)$, which is a generalization of a result of Liu.

## Introduction and main result

The complex oscillation theory of meromorphic solutions of differential equations is an important topic in complex analysis. Some results can be found in Yi and Yang (2003), where Nevanlinna theory is an effective research tool. Recently, many results on meromorphic solutions of difference equations have been rapidly obtained. In this note, we are interested in the properties of entire solutions of difference and differential-difference equations.

Before proceeding, we spare the reader for a moment and assume some familiarity with the basics of Nevanlinna theory of meromorphic functions in ${\mathbb {C}}$ such as the first and second main theorems, and the usual notations such as the characteristic function *T*(*r*, *f*), the proximity function *m*(*r*, *f*) and the counting function *N*(*r*, *f*). *S*(*r*, *f*) denotes any quantity satisfying $S(r, f) = o (T (r, f))$ as $r\rightarrow \infty$, except possibly on a set of finite logarithmic measure—not necessarily the same at each occurrence. Let *a*, *f* be meromorphic functions on ${\mathbb {C}}$. *a* is said to be a small function of *f* whenever $T(r, a)=S(r, f)$. *S*(*f*) denotes the family of all the small functions of *f*. $\lambda (f)$ denotes the exponent of convergence of zeros of *f*, $\sigma (f)$ denotes the order of *f*. A differential polynomial of *f* means that it is a polynomial in *f* and its derivatives with coefficients that are small functions of *f*. A differential-difference polynomial of *f* means that it is a polynomial in *f*, its derivatives and its shifts $f(z+c)$ with coefficients that are small functions of *f*.

For a meromorphic function *f* and a set $S\in {\mathbb {C}}$, we define

$$\begin{aligned} E_{f}(S)=\bigcup _{a\in S}\{z|f(z)-a=0,\,{\text {counting multiplicities}}\}. \end{aligned}$$

We say that *f* and *g* share a set *S* counting multiplicities (CM), provided that $E_{f}(S)=E_{g}(S)$.

Recently, there has been a renewed interest in studying meromorphic solutions of differential-difference equations, see Peng and Chen (2013), Yang and Laine (2010) and Zhang and Liao (2011). Xu et al. (2015) considered a general differential-difference equation to obtain the following theorem.

### **Theorem A**

*Consider the nonlinear differential-difference equation*

1 $$\begin{aligned} q(z)f^{n}(z)+a(z)f^{(k)}(z+1)=p_{1}(z)e^{q_{1}(z)}+p_{2}(z)e^{q_{2}(z)} \end{aligned}$$

*where*$p_{1},\,p_{2}$*are two nonzero polynomials*, $q,\,a$*are two nonzero entire functions of finite order*, $q_{1}\,,q_{2}$*are two nonconstant polynomials*, $n\ge 2$*is an integer. Suppose that an entire functionfsatisfies any one of the following two conditions*:

1. $$\lambda (f)<\sigma (f)=\infty$$,  $$\sigma _2(f)<\infty$$;
2. $$\lambda _2(f)<\sigma _2(f)<\infty$$.
  *Thenfcan not be an entire solution of* (1).

After studying Theorem A, we ask whether the conclusion still holds if the condition $\sigma _2(f)<\infty$ is omitted in (1). In the paper, we consider the problem and give an affirmative answer.

### **Theorem 1**

*Suppose that an entire functionfsatisfies the following condition*:

$$\begin{aligned} \lambda (f)<\sigma (f)=\infty . \end{aligned}$$

*Thenfcan not be an entire solution of* (1).

Liu (2009) used the idea of shared set (see Lü and Xu 2008) and studied the uniqueness problem of entire function *f*(*z*) shares a set with its difference shift $f(z+c)$ as follows.

### **Theorem B**

*Letfbe a transcendental entire function of finite order*, *cis nonzero complex number, and let*$a(z)\in S(f)$*be a non-vanishing periodic entire function with periodc*. *If f(z) and f(z+c) share the set*$\{a(z),-a(z)\}$*CM, then f(z) must take one of the following conclusions*:

1. $$f(z)\equiv f(z+c)$$
2. $$f(z)+f(z+c)\equiv 0$$
3. $f(z)=\frac{1}{2}(h_1(z)+h_2(z))$, *where*$\frac{h_1(z+c)}{h_1(z)}=e^{-\gamma }$, $\frac{h_2(z+c)}{h_2(z)}=e^{-\gamma }$, $h_1(z)h_2(z)=a^2(z)(1-e^{-2\gamma })$*and*$\gamma$*is a polynomial*.

Note that the form of conclusion (3) is not similar to (1) and (2). So, it is necessary to further study the problem. In the paper, we consider Theorem B again. Due to the different method of proof we employ, we obtain the following result.

### **Theorem 2**

*Under the conditions of Theorem* B, *then*

(I) $$f(z)\equiv f(z+2c)$$;
(II) $$f(z)+f(z+2c)\equiv 0$$.

### *Examples*

 Below, we provide two examples to show that the cases (I) and (II) occur.

- **(a)** Let $f(z)=e^z$ and $c=2\pi i$. Then for any $a(z)\in S(f)$, we notice that *f*(*z*) and $f(z+c)$ share {$a(z),-a(z)$} and we can easily see that $f(z)=f(z+2c)$. This example satisfies (I) of Theorem 2.
- **(b)** Let $f(z)=\cos \,z$ and $c=\frac{\pi }{2}$. Then for any $a=\frac{\sqrt{2}}{2}$, we notice that *f*(*z*) and $f(z+c)$ share {$a(z),-a(z)$}. Furthermore, we can easily obtain $f(z)+f(z+2c)=0$. This example satisfies case (II) of Theorem 2.

Tang and Liao (2007) considered the entire solutions of a differential equation. Liu and Cao (2013) considered a q-difference analogue of the above differential equation. Liu and Yang (2013) further generalized the result of Tang and Liao (2007) from differential equations to difference equations. They deduced the entire solutions of generalization of Fermat type equation and obtain below result.

### **Theorem C**

*Let*$P,\,Q$*be two nonzero polynomial. If the difference equation*

2 $$\begin{aligned} f(z)^{2}+P(z)^{2}f(z+c)^{2}= Q(z) \end{aligned}$$

*admits a transcendental entire solution of finite order, then*$P(z)\equiv \pm 1$*andQ*(*z*) *reduces to a constant q. Thus*$f(z)=\sqrt{q}sin(Az+B)$, *whereBis a constant and*$A=(4\pi +1)\setminus 2c$, *where k is an integer*.

At the end of the paper, by considering a different proof of Theorem C, we generalize Theorem C from polynomial *P* to small function *P* as follows.

### **Theorem 3**

*Under the conditions of Theorem* C *and suppose thatP*(*z*) *is nonzero small entire function off*, *then the conclusions of Theorem* C *still hold*.

## Some lemmas

In this section, we state some results that we employ in our proofs.

### **Lemma 1**

(Halburd and Korhonen 2006, Theorem 2.1) *Letfbe a meromorphic function with a finite order, and letcbe complex number*, $\delta <1$. *Then*

$$\begin{aligned} m\left( r,\frac{f(z+c)}{f(z)}\right) =o\left( \frac{T(r,f)}{r^\delta }\right) =S(r,f), \end{aligned}$$

*where*$S(r, f )= o(T (r, f ))$*for allroutside of a possible exceptional setEwith finite logarithmic measure*.

### **Lemma 2**

(Yang and Laine 2010, Theorem 2.3) *Letfbe a transcendental entire function*, *Q*(*z*) *is the canonical product offconstructed by the zeros off*. *Then*$\sigma (Q)=\lambda (Q)=\lambda (f)$.

The Hadamard theorem of entire functions of infinite order with $\sigma _2(f)<\infty$ has been proved in Jank and Volkmann (1985). In the following proof, we need to remove the condition $\sigma _2(f)<\infty$. Similar to the proof of the Hadamard theorem, we prove the following result.

### **Lemma 3**

*Letfbe an entire function of infinite order with*$\lambda (f)<\infty$. *Thenfcan be represented as*

$$\begin{aligned} f(z)=Q(z)e^{g(z)}, \end{aligned}$$

*whereQ*(*z*) *is the canonical product offconstructed by the zeros off*, *g*(*z*) *is a transcendental entire function such that*

$$\begin{aligned} \lambda (Q)=\lambda (f)=\sigma (Q),\,\sigma (f)=\sigma (e^{g})=\infty . \end{aligned}$$

### *Proof*

Let

$$\begin{aligned} F(z)=\frac{f(z)}{Q(z)}. \end{aligned}$$

Then *F*(*z*) is entire with a Picard exceptional value 0, and hence $F(z)=e^{g(z)}$, where *g*(*z*) is an entire function.

Since *Q*(*z*) is the canonical product of *f* constructed by the zeros of *f*, then $\lambda (f)=\lambda (Q)$. By Lemma 2, we have $\sigma (Q)=\lambda (Q)=\lambda (f)<\infty$.

Note that $\sigma (Q)<\sigma (f)=\infty$, we have $\sigma (e^g)=\max \{\sigma (Q), \sigma (f)\}=\sigma (f)=\infty$. $\square$

## Proof of Theorem 1

Suppose that *f* is an entire solution of Eq. (1) and satisfying $\lambda (f)<\sigma (f)$. By Theorem A, it is suffice to prove Theorem 1 for the case $\sigma _2(f)=\infty$.

By Lemma 3, we can set

$$\begin{aligned} f(z)=Q(z)e^{g(z)}, \end{aligned}$$

where *Q* is an entire function, *g* is a transcendental entire function such that

$$\begin{aligned} \lambda (Q)=\lambda (f)=\sigma (Q),\,\sigma (f)=\sigma (e^{g})=\infty . \end{aligned}$$

From the condition $\lambda (f)<\infty$, we have $\sigma (Q)<\infty ,\sigma _{2}(Q)=0$. So $\sigma _{2}(f)=\max \{\sigma _{2}(e^{g}),\sigma _2(Q\})=\sigma _{2}(e^{g})=\sigma (g)=\infty$.

Substituting $f(z)=Q(z)e^{g(z)}$ into (1) we obtain that

3 $$\begin{aligned} q(z)Q^{n}(z)e^{ng(z)}+a(z)H(z)e^{g(z+1)}=p_{1}(z)e^{q_{1}(z)}+p_{2}(z)e^{q_{2}(z)}, \end{aligned}$$

where *H*(*z*) is a differential polynomial in $Q(z+1)$ and $g(z+1)$.

Set $G(z)=g(z+1)-ng(z)$, then (3) becomes

$$\begin{aligned} q(z)Q^{n}(z)+a(z)H(z)e^{G(z+1)}=e^{-ng(z)}(p_{1}(z)e^{q_{1}(z)}+p_{2}(z)e^{q_{2}(z)}) \end{aligned}$$

which implies

4 $$\begin{aligned} q(z)Q^{n}(z)+a(z)H(z)e^{G(z+1)}-e^{-ng(z)}(p_{1}(z)e^{q_{1}(z)}+p_{2}(z)e^{q_{2}(z)})=0. \end{aligned}$$

Let $A_{1}=q(z)Q^{n}(z), A_{2}=a(z)H(z), A_{3}=p_{1}(z)e^{q_{1}(z)}+p_{2}(z)e^{q_{2}(z)}$. It is easy to see that $A_1$ and $A_3$ are of finite order. So $A_1$ and $A_3$ are two small functions of $e^{-ng}$, which means that

$$\begin{aligned} T(r,A_{1})=T(r,A_{3})=S(r,e^{-ng}). \end{aligned}$$

Obviously, $T(r,g)=S(r,e^{-ng})$. Note that *H* is a differential polynomial in $Q(z+1)$ and $g(z+1)$, so $T(r,A_{2})=S(r,e^{-ng})$. Rewrite (4) as

5 $$\begin{aligned} A_{2}e^{G(z+1)}=A_{3}e^{-ng(z)}-A_1. \end{aligned}$$

Next we show that $A_{3}\ne 0$. Suppose $A_3 = 0$, then (1) becomes

$$\begin{aligned} q(z)f^{n}(z)=-a(z)f^{(k)}(z+1), \end{aligned}$$

which implies that $nT(r,f)\le T(r,f)+S(r,f)$, a contradiction. Thus, $A_3\ne 0$.

Suppose that $A_{1}\ne 0$. By using the second main theorem and (5), we have

$$\begin{aligned} \begin{aligned} T(r,e^{-ng})&\le N(r,e^{-ng})+N\left( r,\frac{1}{e^{-ng}}\right) +N\left( r,\frac{1}{e^{-ng}-\frac{A_{1}}{A_{3}}}\right) +S\left( r,e^{-ng}\right) \\&\le N\left( r,\frac{1}{A_{2}}\right) +S(r,e^{-ng})\\&=S(r,e^{-ng}), \\ \end{aligned} \end{aligned}$$

which is a contradiction. So $A_{1}=0$, which implies $Q(z)=0$, a contradiction.

Thus, we finish the proof of Theorem 1.

## Proof of Theorem 2

Since *f*(*z*) is an entire function of finite order and *f*(*z*), $f(z+c)$ share the set {$a(z),-a(z)$}, then,

6 $$\begin{aligned} \frac{(f(z+c)-a(z))(f(z+c)+a(z))}{(f(z)-a(z))(f(z)+a(z))}=e^{\alpha }, \end{aligned}$$

where $\alpha$ is a polynomial. Since *a* is a periodic entire function with period *c*, we infer by Lemma 1 that

7 $$\begin{aligned} m\left( r,\frac{f(z+c)-a(z)}{f(z)-a(z)}\right) =S(r,f) \end{aligned}$$

and

8 $$\begin{aligned} m\left( r,\frac{f(z+c)+a(z)}{f(z)+a(z)}\right) =S(r,f). \end{aligned}$$

From (6)–(8), we obtain

9 $$\begin{aligned} T(r,e^{\alpha })=m(r,e^{\alpha })=S(r,f). \end{aligned}$$

Let $F(z)=f^{2}(z)$, then (6) can be rewritten as

$$\begin{aligned} F(z+c)-a^{2}(z)=(F(z)-a^{2}(z))e^{\alpha }, \end{aligned}$$

which implies

10 $$\begin{aligned} F(z+c)=e^{\alpha }(F(z)-a^{2}(z))+a^{2}(z). \end{aligned}$$

Dividing (10) with $e^{\alpha }$, we get

11 $$\begin{aligned} \frac{1}{e^{\alpha }}F(z+c)=F(z)-a^{2}(z)(1-e^{-\alpha (z)}), \end{aligned}$$

that is, all zeros of $F(z+c)$ are the zeros of $F(z)-a^{2}(z)(1-e^{-\alpha (z)})$. Since *F*(*z*) just has multiple zeros, we have $F(z)-a^{2}(z)(1-e^{-\alpha (z)})$ just has multiple zeros.

Rewrite (10) as

$$\begin{aligned} e^{\alpha }F(z)=F(z+c)-a^{2}(z)(1-e^{\alpha (z)}), \end{aligned}$$

which implies

12 $$\begin{aligned} e^{\alpha (z-c)}F(z-c)=F(z)-a^{2}(z-c)(1-e^{\alpha (z-c)})=F(z)-a^{2}(z)(1-e^{\alpha (z-c)}). \end{aligned}$$

So $F(z)-a^{2}(z)(1-e^{\alpha (z-c)})$ has multiple zeros.

From (9)–(12), it follows that *F*(*z*), $F(z)-a^{2}(z)(1-e^{-\alpha (z)})$ and $F(z)-a^{2}(z)(1-e^{\alpha (z-c)})$ just have multiple zeros.

Suppose that the three functions 0, $a^{2}(z)(1-e^{-\alpha (z)})$ and $a^{2}(z)(1-e^{\alpha (z-c)})$ are distinct from each other.

By using the second main theorem, we obtain

$$\begin{aligned} \begin{aligned}&2T(r,F)\\ & \quad \le \overline{N}\left( r,\frac{1}{F}\right) + \overline{N}\left( r,\frac{1}{F-a^{2}(z)(1-e^{-\alpha (z)})}\right) + \overline{N}\left( r,\frac{1}{F-a^{2}(z)(1-e^{\alpha (z-c)})}\right) + S(r,F)\\&\quad \le \frac{1}{2}N\left( r,\frac{1}{F}\right) + \frac{1}{2}N\left( r,\frac{1}{F-a^{2}(z)(1-e^{-\alpha (z)})}\right) + \frac{1}{2}N\left( r,\frac{1}{F-a^{2}(z)(1-e^{\alpha (z-c)})}\right) \\ & \quad \quad + S(r,F)\le \frac{3}{2}T(r,F)+S(r,F),\\ \end{aligned} \end{aligned}$$

a contradiction.

Then two of the above three functions must be equal.

(i) If $a^{2}(z)(1-e^{-\alpha (z)})=0$, then $e^{\alpha (z)}=1$, which implies $f(z) \equiv f(z+c)$ or $f(z)+f(z+c) \equiv 0$. Furthermore, it leads to the case (I).
(ii) If $a^{2}(z)(1-e^{\alpha (z-c)})=0$, then $e^{\alpha (z-c)}=1$, which implies $e^{\alpha (z)}=1$, we get the same conclusion of (i).
(iii) If $a^{2}(z)(1-e^{-\alpha (z)})=a^{2}(z)(1-e^{\alpha (z-c)})$, then
  $$\begin{aligned} 1-e^{-\alpha (z+c)}=1-e^{\alpha (z)}, \end{aligned}$$
  which implies that $1=e^{\alpha (z)+\alpha (z+c)}$. Then, a calculation leads to $\alpha$ is a constant and $e^{2\alpha }=1$. So, $e^\alpha =\pm 1$.
  If $e^{\alpha }=1$, then we get the same conclusion of (i) and (ii).
  If $$e^{\alpha }=-1$$, then
  $$\begin{aligned} f^{2}(z+c)-a^{2}(z)=-f^{2}(z)+a^{2}(z). \end{aligned}$$
  Furthermore,
  $$\begin{aligned} \begin{aligned} f^{2}(z+2c)-a^{2}(z)&=f^{2}(z+2c)-a^{2}(z+c)=-f^{2}(z+c)+a^{2}(z+c)\\&=-f^{2}(z+c)+a^{2}(z)=f^2(z)-a^{2}(z), \end{aligned} \end{aligned}$$
  which implies $f^{2}(z)=f^{2}(z+2c)$. We obtain $f(z)\equiv f(z+2c)$ or $f(z)+f(z+2c)\equiv 0$, which is (I) or (II).
  Thus, we finish the proof of Theorem 2.

## Proof of Theorem 3

Suppose that *f* is a transcendental entire solution of finite order of (2). Set

13 $$\begin{aligned} G(z)=f^{2}(z),\,H(z)=P^{2}(z)f^{2}(z+c), \end{aligned}$$

then (2) can be rewritten as $G(z)+H(z)=Q(z)$, which implies

14 $$\begin{aligned} G(z)-Q(z)=-H(z). \end{aligned}$$

Thus all the zeros of *H*(*z*) are the zeros of $G(z)-Q(z)$. Since *H* just has multiple zeros, $G-Q$ just has multiple zeros.

From (13), we have $H(z-c)=G(z)P^{2}(z-c)$. Then

15 $$\begin{aligned} \begin{aligned} -G(z-c)=H(z-c)-Q(z-c)&=G(z)P^{2}(z-c)-Q(z-c)\\&=P^{2}(z-c)\left[ G(z)-\frac{Q(z-c)}{P^{2}(z-c)}\right] .\\ \end{aligned} \end{aligned}$$

Note that $G(z-c)=f^2(z-c)$ just has multiple zeros. Then, it follows from the above equation that $G(z)-\frac{Q(z-c)}{P^{2}(z-c)}$ just has multiple zeros.

From (13)–(15), we obtain *G*, $G(z)-Q(z)$, $G(z)-\frac{Q(z-c)}{P^{2}(z-c)}$ just have multiple zeros.

Suppose that the three functions 0, *Q*(*z*), $\frac{Q(z-c)}{P^{2}(z-c)}$ are distinct from each other. Then, by using the second main theorem, we obtain

$$\begin{aligned} \begin{aligned} 2T(r,G)&\le \overline{N}(r,\frac{1}{G})+ \overline{N}\left( r,\frac{1}{G-Q(z)}\right) + \overline{N}\left( r,\frac{1}{G-\frac{Q(z-c)}{P^{2}(z-c)}}\right) + S(r,G)\\&\le \frac{1}{2}N\left( r,\frac{1}{G}\right) + \frac{1}{2}N\left( r,\frac{1}{G-Q(z)}\right) +\frac{1}{2}N\left( r,\frac{1}{G-\frac{Q(z-c)}{P^{2}(z-c)}}\right) + S(r,G)\\&\le \frac{3}{2}T(r,G)+S(r,G),\\ \end{aligned} \end{aligned}$$

a contradiction. Then two of the above three functions must be equal.

Since $Q(z)\ne 0,P(z)\ne 0$, we have $Q(z)=\frac{Q(z-c)}{P^{2}(z-c)}$, which implies

$$\begin{aligned} P^{2}(z)Q(z+c)=Q(z). \end{aligned}$$

Because *Q*(*z*) is a nonzero polynomial, we have $P^2(z)\equiv 1$ and *Q*(*z*) reduces to a constant. Furthermore, by Liu et al. (2012, Theorem 1.1), we obtain the desired result.

Thus, we finish the proof of Theorem 3.

## Conclusions

This paper provides three results. Firstly, we consider the existence of the solutions of a nonlinear differential-difference equation under a general condition. Secondly, we prove a uniqueness theorem of entire function *f*(*z*) shares a set with its difference shift $f(z+c)$. At last, we obtain the entire function solutions of a general Fermat type equation. The above three results were obtained by the different proofs, which can be used later.
